# Assessment of Classification Models and Relevant Features on Nonalcoholic Steatohepatitis Using Random Forest

**DOI:** 10.3390/e23060763

**Published:** 2021-06-17

**Authors:** Rafael García-Carretero, Roberto Holgado-Cuadrado, Óscar Barquero-Pérez

**Affiliations:** 1 Department of Signal Theory and Communications and Telematics Systems and Computing, Rey Juan Carlos University, 28935 Mostoles, Spain; rgcarretero@salud.madrid.org (R.G.-C.); r.holgado.2016@alumnos.urjc.es (R.H.-C.); 2 Deparment of Internal Medicine, Mostoles University Hospital, 28935 Mostoles, Spain

**Keywords:** non-alcoholic fatty liver disease, random forest, interpretability

## Abstract

Nonalcoholic fatty liver disease (NAFLD) is the hepatic manifestation of metabolic syndrome and is the most common cause of chronic liver disease in developed countries. Certain conditions, including mild inflammation biomarkers, dyslipidemia, and insulin resistance, can trigger a progression to nonalcoholic steatohepatitis (NASH), a condition characterized by inflammation and liver cell damage. We demonstrate the usefulness of machine learning with a case study to analyze the most important features in random forest (RF) models for predicting patients at risk of developing NASH. We collected data from patients who attended the Cardiovascular Risk Unit of Mostoles University Hospital (Madrid, Spain) from 2005 to 2021. We reviewed electronic health records to assess the presence of NASH, which was used as the outcome. We chose RF as the algorithm to develop six models using different pre-processing strategies. The performance metrics was evaluated to choose an optimized model. Finally, several interpretability techniques, such as feature importance, contribution of each feature to predictions, and partial dependence plots, were used to understand and explain the model to help obtain a better understanding of machine learning-based predictions. In total, 1525 patients met the inclusion criteria. The mean age was 57.3 years, and 507 patients had NASH (prevalence of 33.2%). Filter methods (the chi-square and Mann–Whitney–Wilcoxon tests) did not produce additional insight in terms of interactions, contributions, or relationships among variables and their outcomes. The random forest model correctly classified patients with NASH to an accuracy of 0.87 in the best model and to 0.79 in the worst one. Four features were the most relevant: insulin resistance, ferritin, serum levels of insulin, and triglycerides. The contribution of each feature was assessed via partial dependence plots. Random forest-based modeling demonstrated that machine learning can be used to improve interpretability, produce understanding of the modeled behavior, and demonstrate how far certain features can contribute to predictions.

## 1. Introduction

So-called fatty liver disease, which involves fat deposition in the liver, may have several causes [1]. Although the most widely accepted cause is alcohol consumption, it also occurs in the absence of alcohol abuse, mainly due to the risk factors of obesity, type 2 diabetes mellitus (T2DM), hyperlipidemia, and metabolic syndrome (MS) [2]. This clinical condition is then called nonalcoholic fatty liver disease (NAFLD), and several studies have associated it with obesity and MS due to the lack of alcohol abuse or other forms of injury (drugs, autoimmune diseases, virus, or hemochromatosis) [3]. NAFLD demonstrates a wide clinical spectrum, from a mild elevation of transaminases to a fibrosis that may lead to cirrhosis and hepatocellular carcinoma [4]. It is estimated that 1 billion people have NAFLD, in both developing and developed countries. However, about a quarter of this population will eventually develop nonalcoholic steatohepatitis (NASH), a further step in the natural course of NAFLD [4]. NASH is characterized by inflammation and liver cell damage, with or without fibrosis Lomonaco2021. It is estimated that 2% of adults with NASH may progress to cirrhosis; among these patients, the incidence of primary malignant tumors of the liver reaches 1–2% per year [3].

NASH is particularly prevalent in patients with T2DM and MS, and the American Diabetes Association recommends assessment for NASH in patients with elevated serum levels of transaminases [5]. Indeed, NAFLD progression, whether to NASH or to fibrosis, is more intense in patients with T2DM or MS. The rationale for these recommendations is that fatty liver disease entails greater mortality and morbidity due to cardiovascular causes, as, from a cardiovascular point of view, NAFLD and NASH can be considered part of the hepatic manifestations of MS and thus can increase cardiovascular events [6].

Both NAFLD and NASH can be treated and even reversed with appropriate therapeutic strategies, such as weight loss or the use of certain drugs [7,8]. However, the gold standard for assessing the grade of disease activity is liver biopsy, which is an invasive technique and may not always be practical, convenient, or even available in certain clinical settings [9]. Given these limitations, routine screening should be centered on clinical features, serum biomarkers [10,11], and what can be obtained from certain noninvasive liver-related techniques, such as elastography (which determines liver stiffness) or ultrasonic attenuation [12]. These noninvasive techniques generally correlate with the grade of fibrosis. However, they are not available in all clinical settings, and there is little information available on patients at a high risk for cardiovascular disease (MS, T2DM, obesity) in outpatient clinics [13,14,15].

Our objective was to develop a classification model based on clinical features and laboratory biomarkers to accurately predict NASH in individuals who are attending a general internal medicine outpatient setting. By developing this model, we hoped to identify the most relevant features for use as therapeutic targets in these patients.

We applied the random forest (RF) algorithm and other tools to assist the interpretation of RF [16]. Predictive models based on machine learning algorithms can have issues when applied to health-related data because of the heterogeneous nature of patients and diagnoses or because clinicians cannot explain predictions in a meaningful way. Then, a complex machine learning-based model can become problematic. In this study, we applied tools to support interpretability. The first tool was to compute feature importance by permuting the values of each feature one by one and checking how this changed model performance. Feature importance is a useful tool for analyzing what features are most important for an overall RF model. Another approach we adopted was to use partial dependence plots (PDPs), which connect the direct relationship between a class label and one or more features of interest. A PDP isolates to what degree the changes made in predictions come from one or another specific feature. A PDP can compute a graphical depiction (plot) of the marginal effect of a feature on the class label [17,18]. Finally, we analyzed individual observations using strategies such as following the prediction path for a single observation to a predicted class, which functions by gathering contributions for a given prediction from each node [19].

### Related Work

The relationship between metabolic syndrome, T2DM, and NAFLD has caught researchers’ attention into new diagnostic strategies. The use of machine learning to classify NASH is not new, and has been proposed by several recent publications, since artificial intelligence can bring new insights into patients with liver diseases. Logistic regression, decision trees, RF, eXtreme Gradient Boosting (XGBoost), or k-nearest neighbors (KNN) have been used with electronic health records (EHR), while neural networks and deep learning have been used for histology and images. Indeed, current machine learning approaches have identified T2DM as a strongly correlated feature with some degree of liver fibrosis and adverse hepatic outcomes (cirrhosis, malignancy) [20]. In addition, machine learning-based predictions are probably more accurate compared with traditional statistical approaches [21]. Most of the previous studies found on medical literature focused on bivariate analyses and developed logistic regression models to find associations between factors causing NASH and the disease. In machine learning-based models, studies aimed to distinguish patients with NASH from healthy individuals. These machine learning models allow researchers to include any number of clinical, laboratory, and demographic features to detect hidden patterns for disease classification. While an extensive revision of literature is beyond the scope of our current study, a recent publication [22] reviewed and discussed advantages and disadvantages of the most frequently used algorithms and other aspects of data (both EHR and imaging). Sowa et al. [23] included EHR of 126 patients to develop a final model with an accuracy of 0.79. However, this model relied on features that are not easily collected or measured, such as apoptosis markers. However, some other machine learning-based strategies have demonstrated a high sensitivity rate for predicting NASH or NAFLD from easily collectable EHR.

Suresha et al. [24] compared several models (logistic regression, RF, and XGBoost), but, according to their results, recurrent neural networks (RNN) achieved the best performance and highest accuracy. Yip et al. [25] included 922 patients to compare logistic regression, AdaBoost, and ridge regression. Finally, the logistic regression model achieved an accuracy of 87–88% and six relevant features, such as insulin resistance, triglycerides, or alanine aminotransferase. Cheng et al. [26] developed several models using KNN, RF, and support vector machines (SVM) to detect NAFLD. They observed that SVM had 86.9% accuracy in men, and RF had 80% in women. Both models selected some relevant features, including cholesterol-related and insulin resistance-related factors. Other algorithms, such as decisions trees, were successfully used by Birjandi et al. [27] for classification of NAFLD.

Fialoke et al. [28] used EHR databases to gain insight into NAFLD. Their study used supervised machine learning to correctly identify patients with NASH using 23 features. They compared performance metrics produced from several models: logistic regression, decision trees, RF, and XGBoost, after preprocessing and cleaning the data. Authors were interested in assessing the contribution of each feature for NASH classification. Although RF produced good performance metrics (accuracy 0.79, precision 0.80, sensitivity 0.76, AUROC 0.87), finally the XGBoost model gave the best performance (accuracy 0.79, precision 0.80, sensitivity 0.77, AUROC 0.87), very similar to RF. When RF was used to rank feature importance, impurity decrease was calculated. It is worth mentioning the study by Doherty et al. [29]. They compared filter methods, KNN, RF, and XGBoost to develop a model for classification of patients with NASH in a cohort of 704 individuals. XGBoost was the best model, with AUROC of 0.82, sensitivity of 0.81, and precision of 0.81. Although the full model included 14 features, a more simplified version included five features with only slightly reduced performance (AUROC 0.79, sensitivity 0.80, and precision 0.80). Nevertheless, most of these studies stressed that their models should not be considered the only diagnostic test, but rather a complementary tool to other tests.

The remainder of this manuscript is structured as follows. In the next section, we introduce the workflow of the data-mining process that we used to collect data and to preprocess them. In addition, we briefly present the RF algorithm and the main interpretability tools used in this research. Following this, we present the results of our experiments and select the final predictive model and the most relevant features. In the final section, we discuss those results and their implications for daily practice for clinicians, and we draw conclusions from them.

## 2. Methods

### 2.1. Data Collection, Exploratory Data Analysis, and Preprocessing

We performed data-mining of electronic health records to build a raw dataset with patients attended at Cardiovascular Risk Unit at Mostoles University Hospital (Madrid, Spain) from 2005 to 2021. All patients had a medical history and a physical examination, from which we collected demographic, anthropometric, and clinical data such as sex, age, height, weight, systolic blood pressure (SBP), and diastolic blood pressure (DBP). Using data from our Department of Clinical Biochemistry, we collected information related to cardiovascular risk, such as levels of cholesterol, fasting plasma glucose (FPG), glycated hemoglobin (HbA1c), blood insulin, and vitamin D. Liver and kidney panels were also included. A Cobas E-601 (Roche Diagnostic, Florham Park, NJ, USA) was used to perform all laboratory analyses. We included 37 independent variables in our dataset (Table 1). T2DM was diagnosed in reference to the American Diabetes Association (ADA) criteria [30]. Insulin resistance was assessed using the homeostatic model assessment of insulin resistance (HOMA-IR), estimated as insulin (mU/L) × FPG (mg/dL)/22.5.

Among our patients, the diagnosis of NASH was based on the appropriate exclusion of inflammatory liver diseases: absence of alcohol consumption (less than 20 g per day), autoimmune hepatitis, viral hepatitis (both B and C hepatitis viruses), Wilson disease, and alpha 1 antitrypsin deficiency. The diagnosis was confirmed via transient elastography using the ultrasound device FibroScan, which assesses fibrosis by measuring the stiffness of the liver. Transient elastography is a painless and noninvasive procedure. Values of F ≥ 2 were considered NASH.

Because we were applying a machine learning classification approach, class labels were considered an outcome. These labels indicate whether or not a patient has been diagnosed with NASH. In terms of types of data, sex was categorical, T2DM and the class label were Boolean, and the remaining 37 features were continuous (numeric), as shown in Table 1.

**Figure 1:**
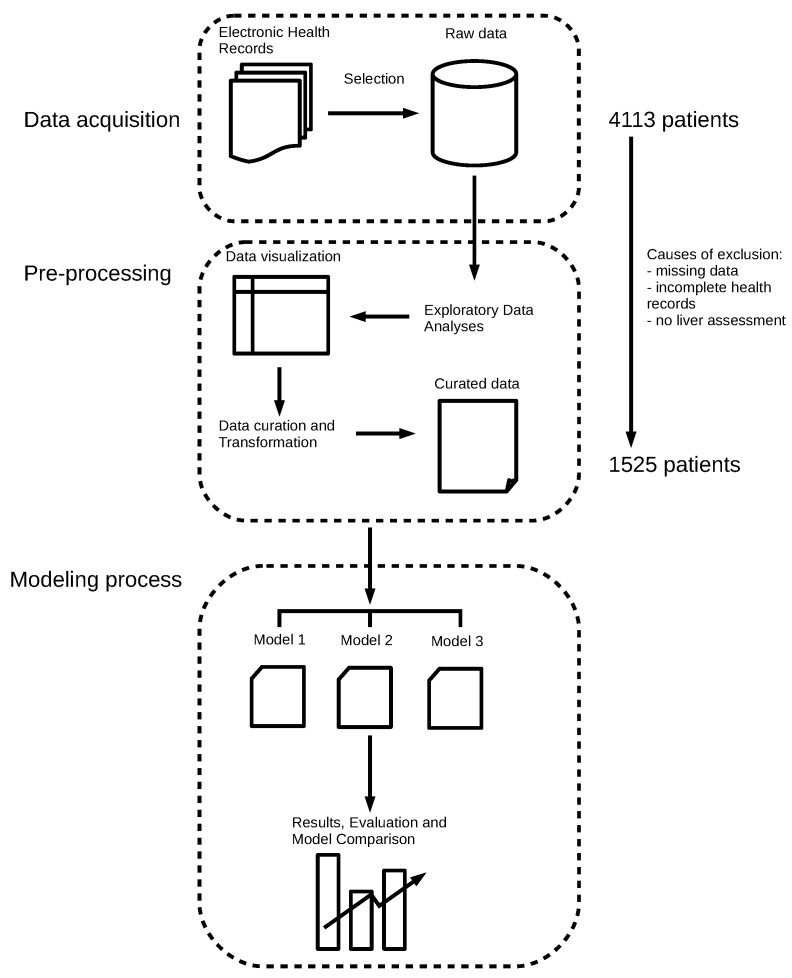
Workflow of the modeling approach used for our dataset.

Next, we explored our dataset to identify aberrant data and outliers. First, the dataset was curated to remove values caused by measurement variability that were nevertheless probable, as well as aberrant data resulting from experimental error. Rows with the latter were reviewed manually to identify the source of error, and, if the values could be amended, the changes were made. If the source of error could not be identified, the whole row of data was discarded. In the final data, no missing values were present. Only 15 rows were excluded because of missing data, and no imputation was needed. Our dataset included real data and no simulations. We consider this to be an opportunity to handle real patients’ data to produce accurate and reliable results. Our objective here was to obtain a well-structured, consistent, and complete dataset, suitable for later analyses. Figure 1 shows the workflow we used to assess our data. A final dataset with 1525 observations was produced.

We also plotted every continuous variable to check its distribution, although normality was also mathematically checked using the Shapiro–Wilk test. Some variables were left-skewed, so a log-transformation was applied, to prevent unstable estimates. We used base-10 logarithm transformation for these variables (Table 1).

Data preparation also involved using standardization to rescale numeric features prior to training a machine learning-based model because non-scaled variables can have a significant impact on model performance or in its interpretability. In our dataset, some features had widely different scales, leading to the risk that those with a wider range would overshadow the others or that small changes in any single feature could significantly change the prediction relative to changes with a narrower range. To make the training task less sensitive to the scales of the predictors, we standardized these ranges so that our final models could be more stable and more reliable. In addition to log-transformation, we also transformed numeric features using the z-score so that they followed a normal distribution, with mean = 0 and standard deviation = 1. The z-score standardization was given by $(x_{i}-\mu )/\sigma$, where $x_{i}$ is the value of the feature, μ is the mean, and σ is the standard deviation.

### 2.2. Class Imbalance

Using real data can be challenging, and health data are more complex than most, as there are unequal instances of the class “disease” because there are usually more healthy individuals than disease cases. In our dataset, the class “no-disease” outweighed the class “disease” at a 3.4:1 ratio. This phenomenon is called data imbalance, and it can have a significant impact on evaluation metrics, such as accuracy, precision, recall, and F1 score. Such metrics may in fact be quite poor in the case of imbalanced classes, as most predictions belong to the majority class. However, there are techniques that can help address this phenomenon and improve prediction performance. We used weighting and sampling techniques because they have the greatest impact on the aforementioned metrics. We applied class weighting, which imposes a heavier cost when errors are made in the minority class. We also used undersampling to select a subset of samples from the class with more instances to match the number of samples coming from each class; that is, we randomly removed instances from the minority class. The main disadvantage here is that we may lose potentially relevant information from the omitted samples. Finally, we applied oversampling to replicate instances in the minority class; that is, we randomly replicated samples from the minority class to bring it to the same size as the majority class. The main disadvantage is that, while it avoids losing information, it may lead to overfitting, i.e., an overestimation of model performance [31,32,33,34,35].

Because we did not know which technique would perform best, we decided to develop several models, attending to the imbalance approach. The first model was a raw-data model, in which features had not been standardized, log-transformed, or resampled. The second was preprocessed, i.e., scaled and log-transformed, but its data were imbalanced. For the third, fourth, and fifth models, we used class weighting, undersampling, and oversampling, respectively, after scaling and log-transforming. The sixth model was a parsimonious model, using the most relevant features.

### 2.3. Data Splitting and Metrics for Performance

After building the five models mentioned, we tested their performance on a testing set. To this end, we split the dataset into two samples with a 3.5 to 1 ratio, i.e., a training sample (70% of the observations) and a testing sample (30% of the observations).

As mentioned, some metrics can be misleading in the context of class-imbalanced datasets. In addition to the use of techniques that can improve performance in such datasets, robust metrics should be adopted. The first such metric is accuracy, defined as the agreement between predicted values and observed outcomes. Mathematically, classification accuracy is expressed as the number of correctly classified individuals (both true positives and true negatives) among the total number of cases. Sensitivity and specificity are related metrics. Sensitivity, or recall, was expressed as the probability of predicting NASH, i.e., the number of true-positive predictions among all positive predictions (both true positives and false negatives). Specificity was expressed as the probability of predicting a disease-free status, i.e., the number of true negatives among all disease-free patients (both true negatives and false positives). Precision shows the probability of having a correct positive prediction, i.e., the number of true positives among all positives retrieved (both true and false positives). The F-measure is calculated from precision and recall, and it provides some balance between these two metrics. The F1 score is used because it does not favor either precision or recall, and it can be calculated as the harmonic mean of both metrics. We also used the area under the receiver operating characteristic (AUROC) curve as a performance metric to evaluate classification models because it computes whether the model is able to correctly rank new samples. We calculated metrics using the following equations:

$$\begin{array}{c} Accuracy=(TP+TN)/TP+TN+FP+FN\\ Precision=(TP)/(TP+FP)\\ Sensitivity=(TP)/(TP+FN)\\ F1score=(2\times TP)/(2\times TP+FN+FP) \end{array}$$

where *TP* is true positive, *FP* is false positive, *TN* is true negative, and *FN* is false negative.

### 2.4. Modeling with the Random Forest Algorithm and Searching for Tuning Parameters

RF is an algorithm based on decision trees, which follows the same principles as a decision tree. A decision tree makes a binary prediction and is simple to implement, but it has very low prediction power. Each node of a decision tree is a decision function. The node child is the potential choice of the previous node. This procedure is performed repeatedly until the tree reaches its end. RF can improve predictions by training a group of decision trees within a random subset of a dataset. By building many decision trees, RF obtains predictions from each individual tree and then predicts the class that obtains the most votes from among the individual trees. In this way, RF generates a wide range of classifiers and aggregates the results. Each node corresponds to a randomly selected feature. In the end, the selected classification results are yielded by the majority of the random decision trees [16,36,37].

In developing an RF model, it is important not to make assumptions, as the algorithm processes data randomly. Finding the best hyperparameters can help obtain better results. In this study, we selected two parameters that we considered would have the largest impact in the model accuracy. The first is the number of randomly selected variables available for splitting at each tree node. The second is the number of trees or branches of a single tree that grow after each split (called mtry and ntree, respectively, following Breiman [36]). Both hyperparameters have a strong influence on the importance estimate for the predictor variable. In addition, a larger number of trees produce more stable models and feature importance estimates but require more computational resources, such as additional memory and a longer run time. For small datasets, 50 trees may be sufficient. For larger datasets, 500 or more might be required [38,39]. Despite the computational cost, we used 5000 trees in all experiments.

We used the packages randomForest [40] and caret [41] from their R language version 3.5.1 [42] to perform our analyses. The package caret allowed us to fine-tune the hyperparameters by means of resampling. Although several methods are available, such as bootstrap or leave-one-out, we used 10-fold cross-validation (10-fold-CV) as the resampling strategy.

### 2.5. Interpretability of Random Forest Models

Correctly interpreting a well-fitted model is as important as obtaining it. A model’s predictions may be accurate, but, most of the time, researchers need to know which features are most predictive. As such, several strategies have been developed to make models more interpretable, to make them white boxes, as opposed to black boxes. Unlike a single decision tree, which is an intuitive model in which each node represents a series of binary queries on the data, eventually leading to a predicted class, RF can yield uninterpretable models, called black boxes [43]. Because a forest consists of a large number of trees, coming to a full understanding of the entire model or even a single tree can be challenging. Furthermore, a single tree can have hundreds or thousands of nodes, so producing an explanatory model is not feasible. Here, we present several approaches to make our model more interpretable.

#### 2.5.1. Importance of Features

Feature importance is one of the most useful tools of interpretation. Moreover, it is only reliable if the model is trained with suitable hyperparameters. The default method for computing feature importance in RF is the mean decrease in impurity mechanism, also called Gini importance, but this strategy can be biased [36]. It is computed by measuring how effective the given feature is at reducing uncertainty in the creation of decision trees within RF. This is a rapid method but can give an inaccurate picture of importance, as it tends to overestimate the importance of some features. Strobl et al. [38] demonstrated that this approach is not reliable when predictors vary in their scale of measurement.

However, RF implementation also provides an alternative technique: permutation importance [44,45]. This approach was introduced by Breiman and Cutler [36,37]. After refolding baseline accuracy by passing a testing set through the model, a single column (i.e., one single feature) is permuted, testing samples are passed back through the model, and accuracy is recomputed. The importance of that permuted feature is the difference between the baseline accuracy and the new computed accuracy after the column is permuted. Obviously, the permutation strategy is more computationally expensive than the Gini method, but the results are more reliable.

In addition to the permutation method, we also used an automatic method for feature selection, called recursive feature elimination (RFE), in which RF is used each iteration to evaluate the model. This method is performed to explore all possible subsets of attributes. When all attributes are used, the resulting plot shows the cross-validation-based accuracy of the different subsets, and, from this, researchers can select a parsimonious model in which a mild loss of accuracy is acceptable.

#### 2.5.2. Partial Dependence Plots

PDPs show the marginal effect of each individual predictor on response probability. This method was introduced by Friedman [17] to allow easier interpretation of complex machine learning algorithms, such as RF. It is useful to find feature importance, but doing so does not indicate whether the feature positively or negatively affects the final model. PDPs are low-dimensional graphical renderings of predictive functions that make the relationship between the feature and the outcome easier to understand [18].

#### 2.5.3. Contribution of Each Feature to the Predictions

There are several methods that can be used to explain single predictions. Each of these compute feature contributions for single predictions. A prediction can be written down in terms of the changes in the value of a feature along a prediction path. Because each node gives a value from a feature, the prediction is defined as the sum of the feature contributions plus bias. One implementation of this approach is the prediction interpretation algorithm introduced by Saabas [46] and Li [19] for RF models. In an RF model, each prediction can be decomposed into a sum of contributions from each feature, as follows:

$$prediction=bias+feature_{1}\;contribution+\ldots +feature_{n}\;contribution$$

The idea here is that, for each decision (i.e., each node of the tree), there is a path from the root to the leaf. Each decision is conditioned by a particular feature that contributes to the final prediction. This concise definition captures the meaning of the tree in the forest: a path through a set of decision nodes that reaches a prediction, together with the information that is available for each node. We used the breakDown package in R, a tool for decomposition of predictions for several black-box type algorithms, such as RF. It provides a table that shows the contribution of each feature to a final prediction. This table can be plotted in a concise, graphical way [47].

### 2.6. Experimental Setup

Having introduced RF and several strategies to interpret and understand its models, we present our experimental setup. After collecting and cleaning the data, we split the dataset into training (70% of observations) and testing (30%) sets. We first fitted the model to a raw training dataset to establish a starting point. Then, we preprocessed (scaled and log-transformed) imbalanced data, balanced data using undersampling, balanced data using oversampling, balanced data using class weighting, and balanced data using class weighting in a parsimonious model given by RFE. In this way, we produced six models, and all of their metrics were compared, using their results in a testing set (the remaining 30% were not used for training the models). Specificity, sensitivity (recall), precision, accuracy, F1 score, and AUROC were computed.

Next, we chose the best model and plotted feature importance. For this, we followed two approaches. The first was to rank features using the permutation method. The other was to use RFE with cross-validation. As noted above, the idea behind the latter is that we cannot know in advance how many features are valid. Therefore, selecting only the top features and achieving a parsimonious model, RFE can allow us to find the optimal number of features through cross-validation to score different feature subsets and select the best scoring collection of features. In this way, a parsimonious model can be developed.

Finally, with the most relevant features selected with RFE, we analyzed the contribution of each feature to the model using PDPs. In addition to these global interpretation approaches, we also explored a method for exploring single predictions by analyzing the contribution of each feature to them (by decomposing the predictions).

## 3. Results

### 3.1. Descriptive Analyses

The final dataset included 1525 patients. Table 2 shows the main clinical characteristics of our cohort. The prevalence of NASH among our participants was 33.2%. Women represented 50.8% of all individuals but were more common in the NASH group, with 57.4% of the cases. A simple bivariate analysis revealed that most features were associated with NASH, including sex, age, T2DM, dyslipidemia, insulin resistance, and hypertransaminasemia. We used filter methods (the chi-square and Mann–Whitney–Wilcoxon tests) to evaluate the relevance of the features because this approach is fast, independent from the classifier, and allows the researcher to ignore irrelevant features. However, because these methods ignore interactions among variables and the classifier (RF, in our case), predictor dependencies could also be ignored. These methods could not identify a relationship among predictors and were not useful for developing our model.

### 3.2. Modeling and Predicting with Random Forest

**Figure 2:**
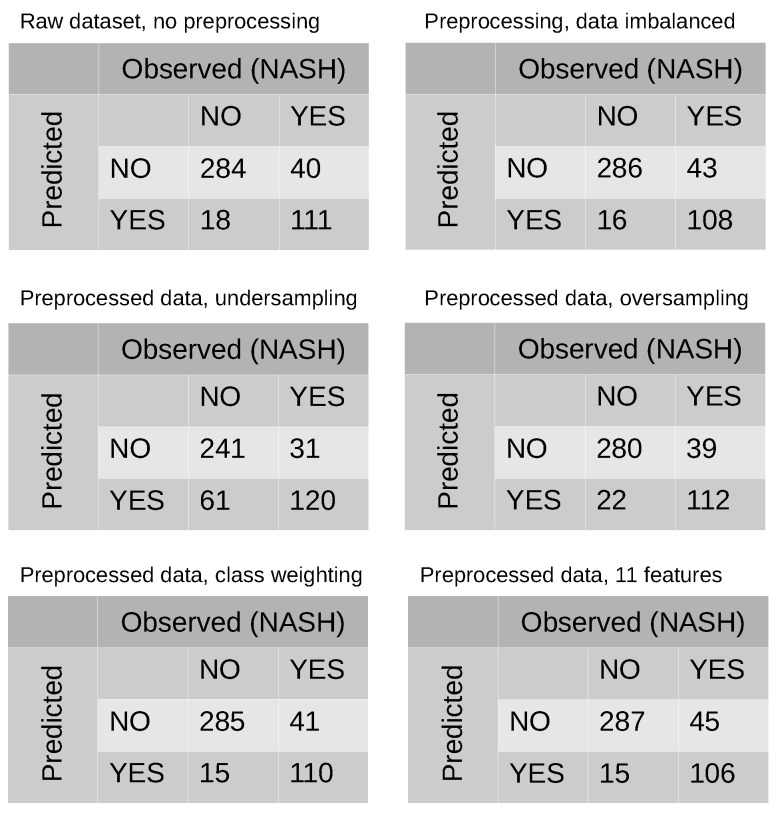
Confusion matrices of every single computed model.

**Figure 3:**
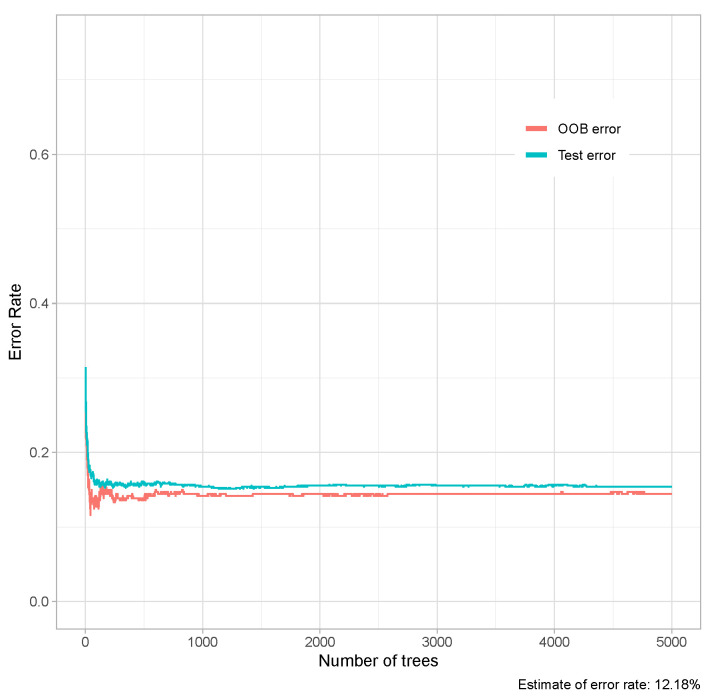
Out-of-bag (OOB) error rate to assess the quality of random forest prediction in both training and testing data sets, shown as a function of the number of decision trees generated during machine learning.

Table 3 shows the six developed models. The first model was a complete dataset, without dropping variables, with no scaling and no transformation. We used 10-fold-CV as a resampling method in RF and grid search to evaluate all of the combinations of mtry in the searching space using cross-validation. Despite there being no preprocessing strategy, it achieved a high prediction score. The next experiment consisted of fitting RF on a scaled, log-transformed but imbalanced dataset. Its performance was similar to the previous model. From that point forward, we perform the experiments after addressing data imbalance. Our first approach was to use class weighting to handle imbalance, and it showed good performance. Next, we used undersampling to handle imbalance. Although its performance was the worst among our experiments, the results obtained were quite good. Finally, we fitted RF using oversampling, and its performance metrics were as good as the previous methods. Confusion matrices for each model are shown in Figure 2. Additionally, we plotted the out-of-bag (OOB) score as a way of validating the RF model (Figure 3).

**Figure 4:**
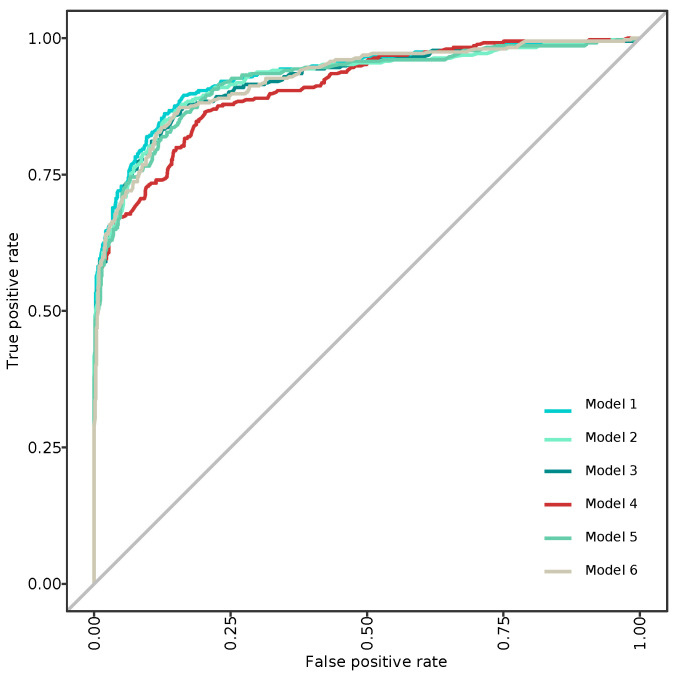
Receiver Operating Characteristic (ROC) curves for every single computed model. Model 1: No preprocessing method; Model 2: preprocessed, but no imbalance strategy; Model 3: Preprocessed, class weighting method; Model 4: Preprocessed, undersampling method; Model 5: Preprocessed, oversampling method; Model 6: Preprocessed, class weighting, parsimonious model.

**Figure 5:**
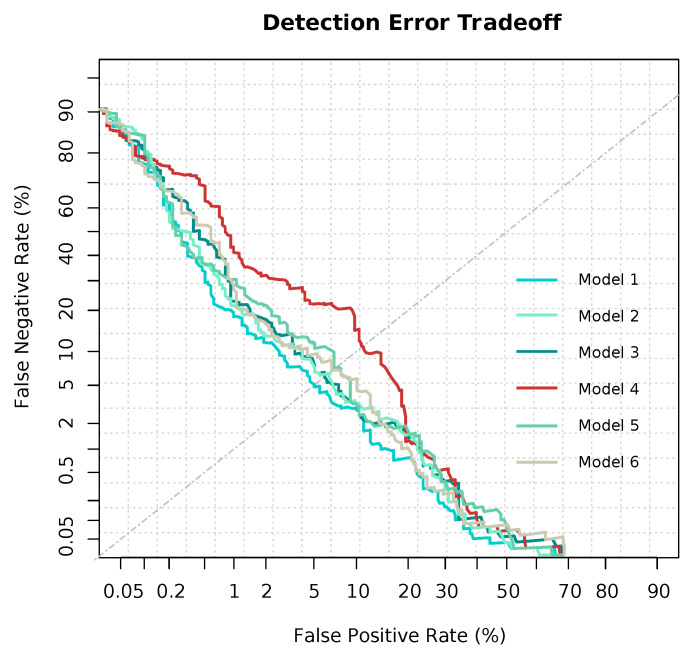
Detection Error Tradeoff (DET) curves of every single computed model. Model 1: No preprocessing method; Model 2: preprocessed, but no imbalance strategy; Model 3: Preprocessed, class weighting method; Model 4: Preprocessed, undersampling method; Model 5: Preprocessed, oversampling method; Model 6: Preprocessed, class weighting, parsimonious model.

ROC curves can seen in Figure 4. We also included Detection Error Tradeoff curves (DET), which are a graphical plot of error rates for binary classification, showing the false rejection rate versus false acceptance rate. It is worth noting that both *x*- and *y*-axes are scaled nonlinearly, so DET curves are more linear than traditional ROC curves, as seen in Figure 5. Model 4, i.e., the preprocessed, undersampling model, showed a significant lower performance than that remaining. On the contrary, the rest of the models showed similar performance metrics, as mentioned in Table 4. Equal Error Rate (EER), as performance metrics, was computed for every model, and shown in Table 3.

Based on Table 3, we cannot conclude that the performance metrics of a certain model were better than the performance of another model on a visual interpretation alone, so we calculated the significance levels using pairwise comparison. Table 4 shows the statistical analyses that were performed to assess the differences among the predictive models. Pairwise comparison using the method of Delong et al. [48] showed no differences among the models, except for the undersampling model, which showed a significantly lower performance.

### 3.3. Importance of Features

**Figure 6:**
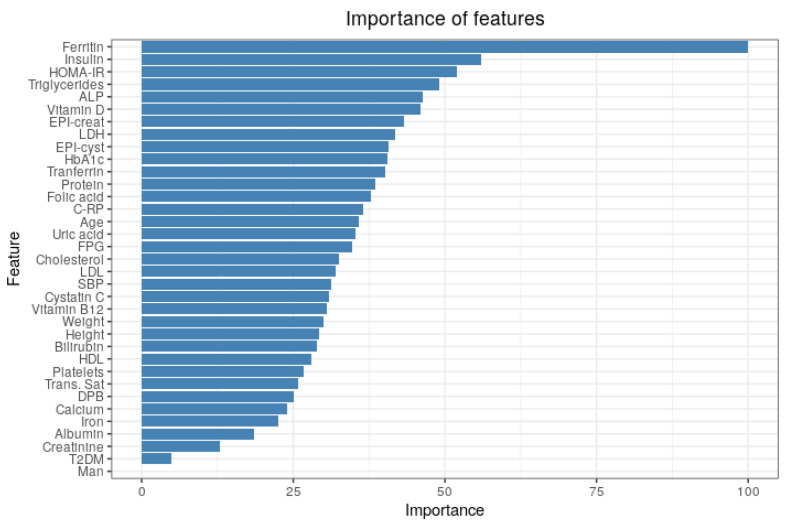
Importance of features based on permutation method using all features.

We selected the class-weighting model to compute feature importance and RFE. As stated previously, we used the permutation method for this. In short, this method works by permuting the values of each feature one by one and establishing how this changes model performance. Figure 6 presents the permutation feature importance, but only the top 20 features are displayed. Here, the serum level of iron was the least important feature, so the remaining variables, which are not displayed, have no relevance in the model.

**Figure 7:**
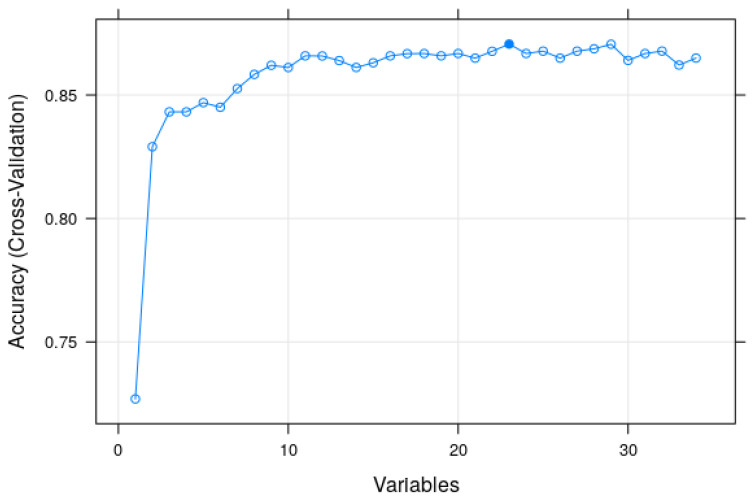
Plot showing accuracy of the model based on the number of features.

RF also allows the researcher to perform RFE [49], as noted. The plot in Figure 7 displays the number of features in the model and their cross-validated test scores and variability, visualizing the selected number of features. The most accuracy was achieved with 24 features. However, because our aim was to obtain a parsimonious model, we trained the model with the top 11 features, which involved only a minor loss of accuracy. The performance of this model is described in the last column of Table 3, and the resultant confusion matrix is shown in Figure 2.

**Figure 8:**
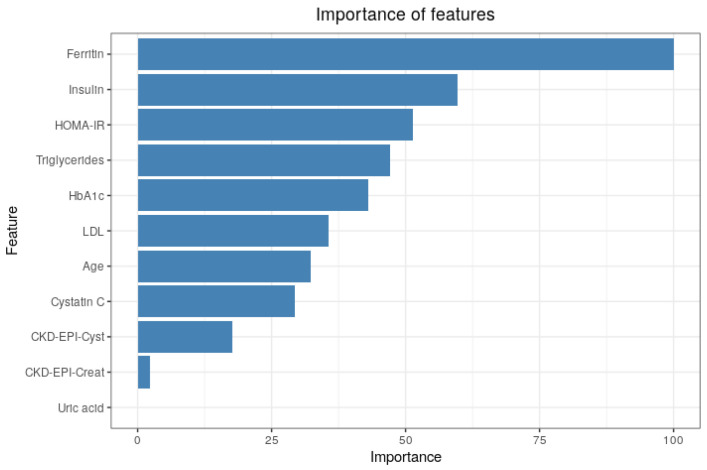
Importance of features based on a permutation method using a parsimonious model with eleven features.

We plotted the model that resulted from using the top 11 features selected from the RFE algorithm, as shown in Figure 8. The top four features matched in both plots. This strategy allowed us to select ferritin, serum levels of insulin, HOMA-IR, and triglycerides as the four most important features in the final model.

### 3.4. Partial Dependence Plots

**Figure 9:**
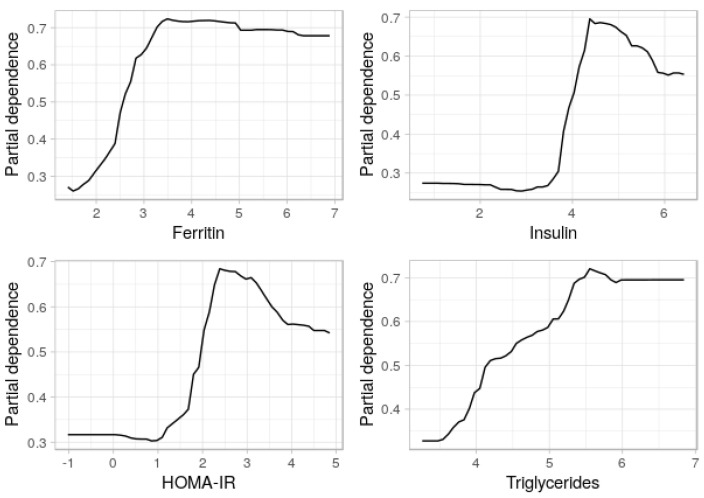
Partial dependence plots of NASH probability and the risk factors ferritin, insulin, HOMA-IR, and triglycerides. Horizontal axis (*x*-axis) shows log-transformed, scaled values. All four variables show a rising trend: the higher their values, the greater the probability of correctly classifying the positive class (here, NASH).

**Figure 10:**
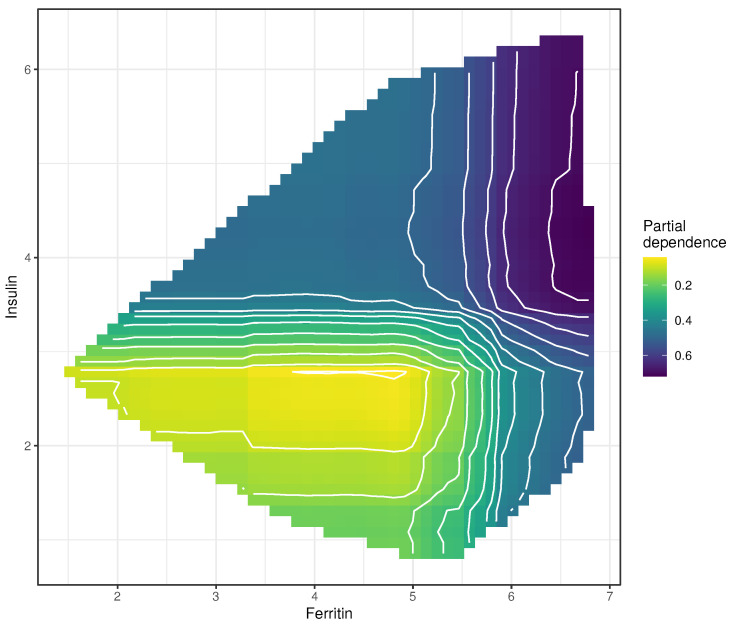
Partial dependence of ferritin and insulin plotted on a probability scale; in this case, the probability of correctly diagnosing NASH.

As indicated, PDPs show how each variable affects the model’s predictions. Thus, next, we selected the parsimonious 11-featured model to generate a PDP. We individually plotted the four most relevant features in our selected model (Figure 9), as well as ferritin and insulin for two-variable analyses (Figure 10). In Figure 9, the *y*-axis does not show the predicted value but the impact of changing the value of the given feature in the positive class, i.e., the odds of having NASH. To take ferritin as an example: we note that the *x*-axis displays a log-transformed, scaled magnitude and not the actual measurements of ferritin. The plot predicts the impact of the values 2, 3, and so on, on the model. PDP computes multiple observations, so the plots display the average predicted class along the vertical axis (*y*-axis). For ferritin and triglycerides, the slope is moderate and smooth, while, for insulin and HOMA-IR, the slope is steep at a certain value, probably because a point is reached where insulin resistance becomes irreversible, overt T2DM.

We also plotted the partial dependence of two features at once. Figure 10 shows the interaction between ferritin and insulin (the two most relevant features, according to our fitted model) with the probability of having NASH. Both ferritin and insulin increase the probability of NASH, regardless of the value of the other variable. However, an interesting interaction occurs at higher values of both variables, namely, a summation effect on the probability of correctly predicting NASH.

### 3.5. Contribution of Each Feature to the Predictions

**Figure 11:**
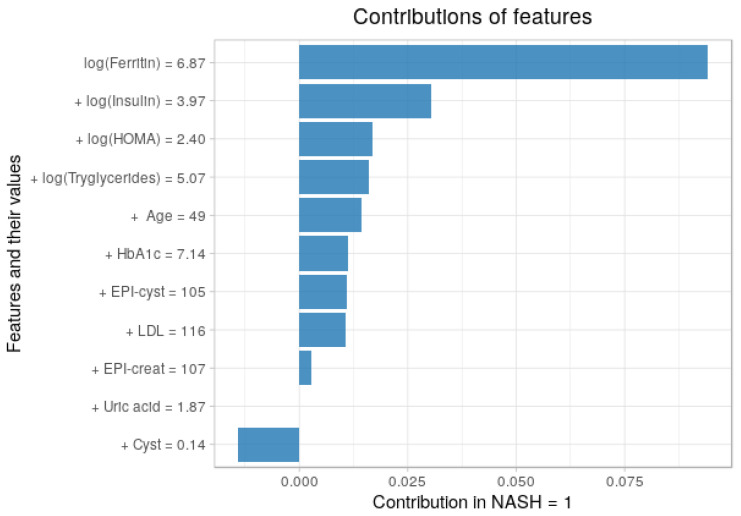
Contributions of features to a final prediction for disease (NASH = 1), showed in terms of both values and contribution.

**Figure 12:**
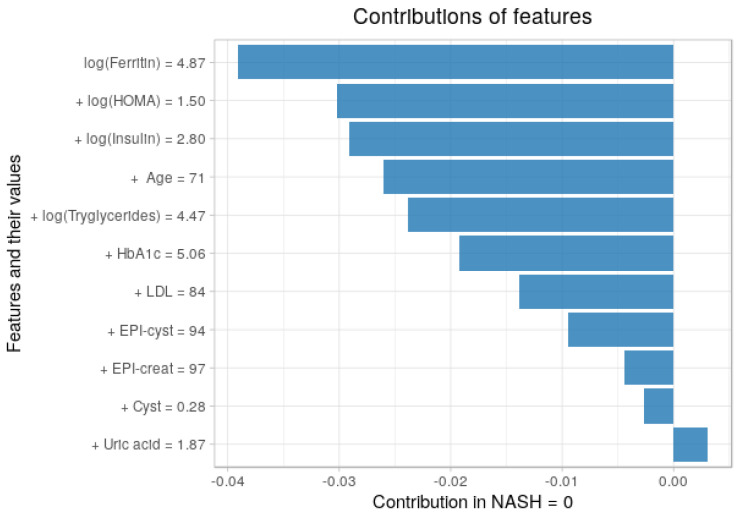
Contributions of features to a final prediction for non-disease (NASH = 0), showed in terms of both values and contribution.

Using the breakDown package in R, we plotted the average contribution in Figure 11 and Figure 12 We analyzed the contribution of each feature in each class (NASH = 1 and for NASH = 0), so its use is based on individual observations or instances. A positive value indicates that the given variable contributes to the correct classification of the disease. A negative value indicates that the probability of a correct classification decreases. It is interesting to observe the behavior of the model in relation to predictions. The pattern of behavior of the model and the features of the four groups presented is quite clear. Values for triglycerides, uric acid, or kidney biomarkers are the least relevant of the eleven selected features and have only a mild impact on predictions. HOMA-IR and insulin usually predict a positive class. Ferritin, however, is very positive in NASH = 1, indicating that it has a significant effect on predictions.

## 4. Discussion

This study investigated physiological phenomena in relation to NASH using predictive models created with RF. RF provides several tools for interpreting the results, such as indicating the importance of variables, the interactions among them, and contribution of each variable to the prediction of NASH.

We chose RF not only because it is one of the most accurate learning algorithms available but also because it is easy to implement. Several methods have been developed to enable its interpretability. It can handle many predictors or features and provides estimates of the importance of different predictors using several methods (including Gini importance and permutation importance). It requires no formal distributional assumptions because RF is non-parametric and can deal with skewed and multi-modal data, as well as categorical and continuous data [16,36,37]. We believe that this is why the model with raw data showed such good performance. Despite these results, no single setting can be reliable, and several settings must be tested to choose the best model for a given scenario. Although hyperparameters go beyond the scope of this research, it is worth noting that conflicting evidence is reported in scientific literature on the importance of mtry values. Some authors have found that mtry does not affect the classification of the RF model or performance metrics because these remain stable under different values of mtry [37]. However, other authors have reported a significant influence of mtry on feature importance [39]. In our study, we developed five models with different mtry values, ranging from 5 to 13. With the exception of the model using undersampling as an imbalance method, the performance of the models was similar in terms of classification, which raises the question of how the different values of mtry actually affect the classification accuracy. In any case, RF is a robust classification algorithm because it can handle left-skewed, non-scaled data while retaining good performance.

In our experiments, we selected an RF model computed with log-transformed, scaled variables, and data imbalances that were handled with class weighting. All experiments, except those that used undersampling for data imbalances, yielded similar results in terms of performance (Table 3 and Figure 2). Hence, we consider RF to be a versatile, robust algorithm, able to deal with data imbalances and non-scaled, non-transformed variables as mentioned before. This phenomenon does not always occur. We do not know beforehand which algorithm, method, or approach will work better. As a simple example, the filter methods shown in Table 2 did produce insight into interactions, contributions, or even relationships among variables or between single variables and the class. In basic statistics, filter methods are used to perform feature selection, but, in our case, they did not provide useful information. Likewise, we could not make assumptions regarding which method would perform better in RF. In our dataset, when data were not preprocessed, performance was quite good, but if that had been our unique approach, the results would not be reliable, and overfitting should have been suspected. In this research, several strategies were used, but, for the sake of simplicity, we selected only one model for interpreting the results.

We were aware that the RF algorithm is invariant to monotonic transformations of individual features, i.e., per feature scaling will not change results in an RF model. RandomForest is tree based, and, for tree based algorithms, scaling is not required. We decided to scale and log-transform because of two main reasons: (1) while it is not necessary, scaling or log-transforming will not harm the final model; if only predictions are required, then common sense says that scaling is not required. However, if either feature importance or feature selection are under consideration, then scaled vs. unscaled data might give different feature-related results, as pointed out by Strobl et al. [38]. In addition, (2) scaling and log-transforming are healthy habits when using machine learning approaches, and can be valuable both for making patterns in the data more interpretable and for helping to meet the assumptions of inferential statistics. Our preprocessing methods did not hamper the results or the performance of our proposed approaches. Indeed, our models yielded similar results in terms of performance, which helps to demonstrate some of the advantages of RF: it works with both categorical and continuous features, it handles nonlinear parameters, it is robust to outliers, and it is very stable, since results will not be affected regardless of the scale or the skewness.

The use of interpretability tools demonstrated that RF is not a black box and can be made into a white box when the appropriate tools are used. The first tool for interpreting our results was feature importance. We used the permutation strategy instead of the impurity method. A more parsimonious model could be achieved with the feature importance tool. Instead of considering all 37 variables, we selected the most relevant ones using both RFE and the feature importance plot via permutation. The top four features were ferritin, insulin, HOMA-IR, and triglycerides. Permutation importance is a reasonably efficient, reliable method of computing feature importance and usually achieves high performance and interpretability. It directly measures feature importance by observing the effects on model accuracy, after shuffling each feature. Permutation importance can be recommended for RF either for classification or for regression tasks, as it can avoid issues with model parameter interpretation [50]. However, although permutation importance is efficient, Strobl et al. [38,39] showed that it often over-estimates the importance of correlated predictors. In our case, insulin and HOMA-IR are correlated, and we found a risk for potential bias toward these correlated features. This issue can be overcome using the drop-column importance mechanism, but this approach is prohibitively expensive in terms of computing resources, given the high number of features involved. From a clinical point of view, the contribution of a serum level of insulin and HOMA-IR is the same, as we demonstrated that insulin resistance is a key factor in the development of NASH and thus can be used as a therapeutic target.

With the aid of PDP, we could analyze the marginal effects of the four selected features. The greater the amount of ferritin, the greater the odds of having NASH (Figure 9 and Figure 10). The same is true for the other three variables. PDP shows a threshold above which the probability of having NASH increases. After reverse log-transformation and scaling, the average thresholds were 177 mg/dL for ferritin (upper normal limit: 250 mg/dL), 53 mIU/L for insulin (normal value: <25 mIU/L), 2.0 for HOMA-IR (normal range: 0.5–1.4), and 170 mg/dL for triglycerides (normal value: <170 mg/dL).

Using PDP and feature importance, we covered the global interpretability of a model that can be used in a whole dataset of a population with similar demographic and clinical characteristics as our cohort. Local interpretability techniques have a significant advantage for the analysis of the local contribution of every feature in single observations and can help the researcher explain incorrect model predictions for a given individual. We have established both the global and local (individual) interpretability of our model. The modalities are not incompatible, and a researcher should select the technique that best suits a given clinical scenario.

From a clinical point of view, this study builds upon our recent study on NASH and machine learning techniques where least absolute shrinkage and selection operator and RF were adopted to find the most relevant features [11,51]. In this study, we conducted thorough analyses using a variety of methods to improve preprocessing data and tools to better understand and explain our results. We found that, in insulin resistance (determined by either HOMA-IR or serum levels of insulin), high levels of serum ferritin and triglycerides are related to the probability of having NASH. Iron in the form of ferritin is stored in the liver, probably due to mild systemic inflammation, which can include liver inflammation [52]. Ferritin is considered an acute-phase protein, which may be elevated not only in systemic inflammation but also in mild inflammation as induced by metabolic syndrome and T2DM [53,54]. Although it remains unclear whether ferritin is the cause or consequence of NASH, its association with direct injury in the liver has been demonstrated, and the severity of the histological lesions in both NASH and NAFLD has been predicted. Thus, this can be a useful biomarker for identifying patients at a high risk for NASH [55,56].

Both insulin resistance and hypertriglyceridemia are related to liver damage, probably because they can induce mild systemic inflammation and the progression of NAFLD to NASH [57]. Obesity, metabolic syndrome, and insulin resistance play a role in both NAFLD and NASH because dysregulation of several adipokines induced by hyperinsulinemia may lead to local inflammatory processes and liver damage [57,58,59]. Another major clinical implication of our research is the identification of a therapeutic target: if insulin resistance is involved in the development of NASH, the use of hypoglycemic drugs can be useful for preventing NASH, as reported in a recent meta-analysis [60].

### Limitations

The main limitation of our study was the intrinsic characteristics of our population. Our cohort tended to be obese and hypertensive, and many had prediabetes, diabetes, or metabolic syndrome. This high-risk population tends to have a high prevalence of NASH, more than the general population. Although the prevalence identified here is relevant to this cohort, we do not know their true importance for other populations. For instance, a recent work established that the prevalence of NAFLD in a middle-aged population in the United States was 38%, with a NASH prevalence of 14% [61].

## 5. Conclusions

This study highlights the use of complex machine learning algorithms such as RF, several strategies for preprocessing data prior to analyzing them, and several tools for interpreting, understanding, and explaining the results. We developed a reliable and understandable predictive model, demonstrating some optimal techniques for analyzing real data on patients at a high risk for NASH. The performance of this model, in terms of prediction accuracy, AUROC, precision, recall, and F1-score, can be considered good. Features considered the most relevant are consistent with their physiological role. Unlike other alternatives, whether invasive or noninvasive, such as biopsy, elastography, or analysis of serum biomarkers, our noninvasive approach can be used in daily clinical practice, not only to screen patients for liver biopsy [62,63] but also to identify therapeutic targets such as triglycerides and insulin resistance. Furthermore, our main technical contribution was to provide a machine learning strategy to make black box models such as RF more interpretable, while remaining simple and well-fitted for identifying NASH in populations at risk.

## Figures and Tables

**Table 1:** Dataset features.

Feature Name	Type	Measurement Unit	Skewness
Age	numeric	years	no
Sex	categorical	Men, Woman	
Weight	numeric	kg	no
Height	numeric	centimeters	no
Systolic blood pressure (SBP)	numeric	mmHg	no
Diastolic blood pressure (DBP)	numeric	mmHg	no
Vitamin D	numeric	ng/dL	no
Folic acid	numeric	ng/dL	no
Serum albumin	numeric	g/dL	no
Bilirubin	numeric	mg/dL	left-skewed
Serum calcium	numeric	mg/dL	left-skewed
Creatine phosphokinase (CPK)	numeric	mg/dL	left-skewed
Cholesterol	numeric	mg/dL	left-skewed
LDL-cholesterol	numeric	mg/dL	left-skewed
HDL-cholesterol	numeric	mg/dL	left-skewed
Triglycerides	numeric	mg/dL	left-skewed
Alkaline phosphatase (ALP)	numeric	IU/L	left-skewed
Serum iron	numeric	μg/dL	no
Ferritin	numeric	mg/dL	left-skewed
Transferrin saturation percentage	numeric	percentage	no
Gamma-glutamyl transferase (GGT)	numeric	mg/dL	left-skewed
HOMA-IR	numeric	NA	left-skewed
Insulin	numeric	μU/mL	left-skewed
Glycated hemoglobin (HbA1c)	numeric	percentage	left-skewed
Fasting plasma glucose (FPG)	numeric	mg/dL	left-skewed
Lactate dehydrogenase (LDH)	numeric	IU/L	no
Platelet count	numeric	cells per mL	left-skewed
Serum proteins	numeric	g/dL	left-skewed
Uric acid	numeric	mg/dL	left-skewed
Vitamin B12	numeric	ng/L	no
Creatinine	numeric	mg/dL	left-skewed
Cystatin C	numeric	mg/dL	left-skewed
C-reactive protein (CRP)	numeric	mg/L	left-skewed
Body mass index (BMI)	numeric	kg/m^2^	no
CKD-EPI-Creatinine	numeric	mL/min/1.72 m^2^	no
CKD-EPI-Cystatnin C	numeric	mL/min/1.72 m^2^	no
Type 2 diabetes mellitus (T2DM)	Boolean	0.1	
Nonalcoholic steatohepatitis (NASH)	Boolean	0.1	

The class label was NASH (1, “disease” label, or 0, “non-disease” label). If a single feature was left-skewed, it was log-transformed. CKD-EPI-Creat: creatinine-based Chronic Kidney Disease Epidemiology Collaboration filtration rate. CKD-EPI-Cyst: cystatin C-based Chronic Kidney Disease Epidemiology Collaboration filtration rate. HOMA-IR: homeostasis model assessment–insulin resistance. LDL: low density lipoprotein. HDL: high density lipoprotein.

**Table 2:** Demographic and clinical characteristics of our studied cohort.

	Total	With NASH	Without NASH	*p* Value
Patients	1525	507	1018	
Sex (men)	48.9	42.4	52.1	0.2
Age	57.3 ± 13.4	54.8 ± 11.6	58.5 ± 14.0	<0.001
Weight	78.3 ± 15.1	81.0 ± 15.6	77.6 ± 14.9	<0.001
Height	161.3 ± 14.4	162.9 ± 11.0	160.2 ± 30.2	0.3
SBP (mmHg)	146.8 ± 20.0	148.0 ± 19.8	146.2 ± 20.1	0.174
DBP (mmHg)	85.6 ± 11.2	87.6 ± 10.9	84.6 ± 11.2	<0.001
Vitamin D	18.0 ± 6.7	17.9 ± 7.1	18.1 ± 6.5	0.32
Folic acid	14.0 ± 5.9	13.8 ± 6.0	14.2 ± 5.8	0.96
Serum albumin	4.1 ± 0.4	4.1 ± 0.5	4.1 ± 0.4	0.552
Bilirubin	0.8 (0.5)	0.9 (0.4)	0.8 (0.5)	<0.001
Serum calcium	10.2 (0.5)	10.3 (0.6)	10.2 (0.5)	<0.001
CPK	146.0 (118.0)	180.0 (173.5)	143.0 (91.8)	<0.001
Cholesterol	224.3 (40.0)	231.3 (46.0)	220.8 (36.2)	<0.001
LDL	139.0 (32.0)	144.6 (33.5)	136.2 (30.9)	<0.001
HDL	67.8 (18.9)	67.1 (20.5)	68.1 (18.0)	0.29
Triglycerides	167.0 (118.0)	194.0 (138.0	152.0 (104.0	<0.001
ALP	86.0 (32.0)	90.0 (36.5)	84.0 (32.8)	<0.001
Serum iron	56.6 ± 23.3	54.9 ± 21.0	57.5 ± 24.4	0.139
Ferritin	185.0 (176.2)	247.0 (238.0)	162.0 (136.0)	<0.001
Transferrin sat. (%)	13.2 (7.7)	12.5 (8.2)	13.2 (7.5)	0.33
GGT	38.0 (46.0)	63.0 (80.0)	31.0 (29.0	<0.001
HOMA-IR	4.5 (2.9)	5.2 (5.2)	4.5 (1.9)	<0.001
Insulin	16.5 (9.2)	18.5 (16.0)	16.5 (6.3)	<0.001
HbA1c (%)	6.2 (1.0)	6.3 (1.7)	6.2 (0.8)	<0.001
FPG	117.0 (42.0)	127.0 (64.0)	114.0 (36.0)	<0.001
LDH	300.9 ± 86.7	309.2 ± 93.1	296.8 ± 83.0	0.17
Platelet count	288.0 (94.0)	293.0 (85.0)	284.5 (97.0)	0.137
Serum proteins	7.70 (0.5)	7.76 (0.5)	7.6 (0.6)	0.223
Uric Acid	7.2 (2.7)	7.7 (3.5)	6.9 (2.2)	<0.001
Vitamin B12	451.6 ± 152.2	470.0 ± 159.8	442.4 ± 147.4	0.3
Creatinine	0.8 (0.2)	0.8 (0.3)	0.8 (0.2)	0.9
Cystatin C	0.7 (0.2)	0.7 (0.2)	0.8 (0.2)	<0.001
C-RP	8.7 (15.1)	10.8 (15.5)	7.7 (13.9)	<0.001
CKD-EPI-Creatinine	92.8 ± 18.5	97.5 ± 15.8	90.4 ± 19.3	<0.001
CKD-EPI-Cystatin C	101.5 ± 22.5	107.5 ± 20.3	98.6 ± 23.0	<0.001
T2DM (%)	43.3	54.7	37.6	<0.001

Data are shown as means ± standard deviations or as medians (interquartile ranges), where appropriate. Categorical and binary features are expressed as percentages. The results of Mann–Whitney–Wilcoxon tests are given for numeric features, and the results of the chi-square test are given for categorical variables.

**Table 3:** Performance metrics of the five different computed models.

	No Preprocessing	Preprocessing Methods				
		No Imbalance Method	Class Weighting	Undersampling	Oversampling	Parsimonious Model
Accuracy	0.872	0.869	0.872	0.796	0.865	0.867
Sensitivity	0.642	0.715	0.728	0.794	0.741	0.702
Specificity	0.966	0.947	0.943	0.798	0.927	0.950
Precision	0.906	0.869	0.874	0.886	0.877	0.864
F1-score	0.751	0.806	0.807	0.739	0.801	0.805
AUC	0.837	0.831	0.836	0.796	0.834	0.829
EER	0.162	0.168	0.162	0.207	0.165	0.147

**Table 4:** Pairwise comparison of ROC curves.

	No Imbalance Method	Class Weighting	Undersampling	Oversampling	Parsimonious Model
No preprocessing	Z = 0.946 *p*-value = 0.343	Z = 0 *p*-value = 1	Z = 3.170 ***p*****-value = 0.001**	Z = 0.391 *p*-value = 0.695	Z = −1.235 *p*-value = 0.216
No Imbalance Method		Z = −1.070 *p*-value = 0.284	Z = 2.505 ***p*****-value = 0.012**	Z = −0.430 *p*-value = 0.666	Z = −1.870 *p*-value = 0.061
Class Weighting			Z = 3.068 ***p*****-value = 0.002**	Z = 0.537 *p*-value = 0.591	Z = −1.408 *p*-value = 0.160
Undersampling				Z = −2.904 ***p*****-value = 0.003**	Z = −3.601 ***p*****-value = 0.003**
Oversampling					Z = −1.64 *p*-value = 0.101

Z stands for Z-statistics by Delong [48].

## Data Availability

Our project included data from a single institution collected under IRB approval and did not utilize public funding or resources. The IRB approval of our institution clearly stated that the data will remain with the principal investigator (Garcia-Carretero) and the study investigators. We would like to note that, to date, there are many ongoing analyses from this project. Data sharing will only occur on a collaborative basis after the approval of all investigators and our institution. According to the terms of a contract signed with Mostoles University Hospital, the authors cannot provide the dataset to any other researcher. Furthermore, they must destroy the dataset at the conclusion of their work with it.
